# Rapid and Sensitive Identification of the Herbal Tea Ingredient *Taraxacum formosanum* Using Loop-Mediated Isothermal Amplification

**DOI:** 10.3390/ijms16011562

**Published:** 2015-01-09

**Authors:** Guan-Hua Lai, Jung Chao, Ming-Kuem Lin, Wen-Te Chang, Wen-Huang Peng, Fang-Chun Sun, Meng-Shiunn Lee, Meng-Shiou Lee

**Affiliations:** 1 Graduate Institute of Biotechnology, National Chung Hsing University, Taichung 40402, Taiwan; E-Mail: just_dfm@hotmail.com; 2 Department & Institute of Pharmacology, National Yang-Ming University, Taipei 11221, Taiwan; E-Mail: rich720925@yahoo.com.tw; 3 Department of Chinese Pharmaceutical Science and Chinese Medicine Resources, China Medical University, Taichung 40402, Taiwan; E-Mails: linmk@mail.cmu.edu.tw (M.-K.L.); wtchang@mail.cmu.edu.tw (W.-T.C.); whpeng@mail.cmu.edu.tw (W.-H.P.); 4 Department of Bioresources, Da-Yeh University, Changhua 51591, Taiwan; E-Mail: fcsun@mail.dyu.edu.tw; 5 Management Center, Department of Medical Research and Development, Show Chwan Health Care Sysytem, Changhua 51951, Taiwan; E-Mail: mengshiunn@gmail.com

**Keywords:** *Taraxacum formosanum*, medicinal plant, internal transcribed spacers 2 (ITS2), loop-mediated isothermal amplification (LAMP), rapid authentication, adulterant, quality control

## Abstract

*Taraxacum formosanum* (TF) is a medicinal plant used as an important component of health drinks in Taiwan. In this study, a rapid, sensitive and specific loop-mediated isothermal amplification (LAMP) assay for authenticating TF was established. A set of four specific LAMP primers was designed based on the nucleotide sequence of the internal transcribed spacer 2 (ITS2) nuclear ribosomal DNA (nrDNA) of TF. LAMP amplicons were successfully amplified and detected when purified genomic DNA of TF was added in the LAMP reaction under isothermal condition (65 °C) within 45 min. These specific LAMP primers have high specificity and can accurately discriminate *Taraxacum formosanum* from other adulterant plants; 1 pg of genomic DNA was determined to be the detection limit of the LAMP assay. In conclusion, using this novel approach, TF and its misused plant samples obtained from herbal tea markets were easily identified and discriminated by LAMP assay for quality control.

## 1. Introduction

The loop-mediated isothermal amplification (LAMP) assay is a well-developed method capable of rapid and specific DNA amplification. The reaction is catalysed using *Bst* DNA polymerase instead of *Taq* DNA polymerase using four to six specifically designed primers under isothermal conditions [1]. Because of the strand displacement activity of *Bst* DNA polymerase, the LAMP reaction can be performed without the denaturation step of PCR [1,2]. Until now, the LAMP method has been applied to the detection of various microbes or pathogens such as protozoa, bacteria, and viruses [3,4,5,6,7,8,9,10]. Recently, a number of studies have demonstrated that LAMP can be applied to the detection of various plants, including genetically modified organisms (GMOs) [11,12,13]. However, LAMP has yet to be used to authenticate medicinal herbs [14,15,16]. LAMP is time-effective, with high specificity and sensitivity and requires relatively simple equipment; consequently, it has become a very powerful tool for developing a nucleic acid-based diagnostic method for identification or authentication of bio-resources.

*Taraxacum formosanum* (TF) is a traditional Chinese medicine used in Taiwan as well as mainland China. A whole plant extract of *Taraxacum*, also commonly known as herbal tea, is a popular folk drink with a number of functional ingredients and traditionally used by herbalists and doctors for the treatment of boils, sores, inflammation of the eye, urethral infection, lung and breast abscesses, acute appendicitis and jaundice [17]. *Taraxacum* has been found to have antibacterial, antifungal, antileptospiral, and antiviral effects, all of which have been reported by previously studies [17,18,19]. However, since their similar morphological characteristics without the key taxonomic diagnostic features on the plants, *Taraxacum officinale*, *Ixeridium laevigatum*, *Youngia japonica*, *Ixeris chinensis* and *Emilia sonchifolia* var. *javanica* are very commonly misused in place of TF in the herbal medicine market [19,20,21,22]. The use of histological microscopy and chemical profiling by HPLC has been documented in authenticating TF and identifying its adulterants [23]. However, the morphological features and chemical makeup of TF may be affected by environmental or other factors such as growth, harvest time and storage condition [21]. Up to now, DNA level identification has a number of advantages for the authentication of medicinal plants, as they are more effective, reliable, sensitive, and easily standardized compared to other conventional methods [24,25,26,27].

DNA barcoding using a highly variable DNA region is a powerful technique when employed to distinguish organism or species [27]. The internal transcribed spacer (ITS) of nuclear ribosomal DNA (nrDNA) and some non-coding or coding regions of plastid genome such as *rbc*L, *mat*K, *rop*C1, *ycf*5 and *psb*A-*trn*H have been widely used as barcode candidates for plant species identification [28,29,30]. So far, a few molecular DNA techniques, such as Random Amplified Polymorphic DNA (RAPD) analysis and PCR-RFLP have been used to identify or authenticate TF and have allowed this plant to be distinguished from its commonly misused adulterants [4,31]. Nonetheless, the RAPD and RFLP approaches are problematic when it comes to reproducibility and procedural complication. The aim of this study was to employ the highly specific ITS region of TF as a target DNA region for developing a LAMP assay that would allow rapid identification of TF from its adulterants. We successfully established that the LAMP approach is more convenient and can be used as a model for creating a medicinal plant identification method. To our best knowledge this is first report describing development of LAMP for TF identification or authentication.

## 2. Results

### 2.1. Analysis of ITS2 Sequence Alignment

**Figure 1:**
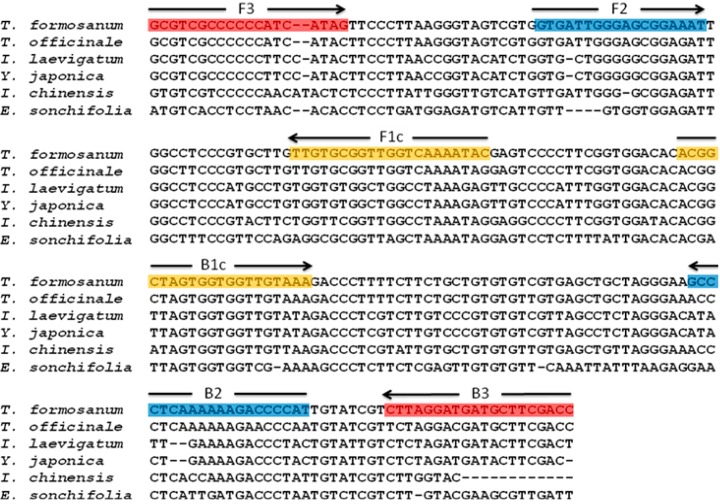
Sequence alignment of the ITS2 of *T. formosanum* and its adulterants. The five ITS2 sequences of the adulterants of *T. formosanum* were aligned against *T. formosanum*. Hyphens (-) denote alignment gaps. The color boxed sequences indicate the regions where the specifically designed LAMP primer sequences are found. The arrow symbols indicate the direction of DNA polymerization from the LAMP primers.

To design the LAMP primers for the authentication of TF, the ITS2 regions of the nuclear ribosomal DNA of TF was used as a target DNA region. The ITS2 sequence of TF and its adulterants, *Taraxacum officinale* (TO), *Ixeridium laevigatum* (IL), *Youngia japonica* (YJ), *Ixeris Chinensis* (IC), *Emilia sonchifolia* var. *javanica* (ES) were obtained from GenBank and aligned (Figure 1). The interspecies variation of ITS2 among TF and its adulterants ranged from 27.02% to 59.45%, which is very useful when designing LAMP primers. Based on the interspecies and intraspecies sequence variation of ITS2, four specific LAMP primers, F3, B3, FIP and BIP, were designed to specifically amplify the TF target DNA under isothermal conditions (Table 1).

**Table 1:** LAMP primer used in this study.

Primer Name	Type	Length (bp)	Sequence (5'-3')
F3	F3	18	GCGTCGCCCCCATCATAG
B3	B3	20	GGTCGAAGCATCATCCTAAG
FIP	F2 + F1c	38	GTATTTTGACCAACCGCAC AAGTGATTGGGAGCGGAAT
BIP	B2 + B1c	41	ACGGCTAGTGGTGGTTGTAAAA TGGGGTCTTTTTTGAGGGC

### 2.2. Development of LAMP Primers for Authentication of T. formosanum

**Figure 2:**
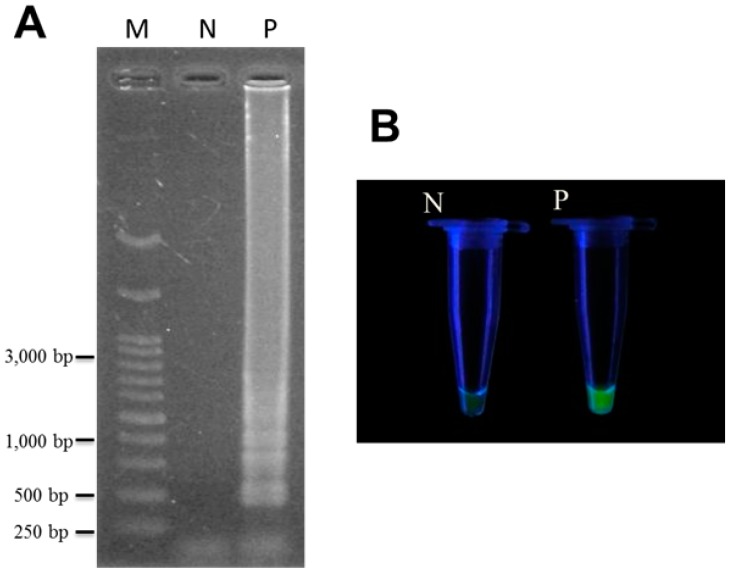
Detection of the LAMP products of *T. formosanum* (TF) using DNA electrophoresis (**A**) and fluorescence (**B**). LAMP reaction was performed with the specifically designed TF LAMP primers. The right panel (B) represented the LAMP products stained with SYBR Green I and visualized under UV excitation. Fluorescence was observed in the samples containing TF genomic DNA. Lane M, 1 kb of DNA ladder marker; lane N, without TF genomic DNA; lane P, with TF genomic DNA.

When the LAMP reaction is performed with these specific designed LAMP primers using extracted TF genomic DNA as a template, the LAMP product showed the presence of specific ladder-like DNA fragments on gel electrophoresis (Figure 2A). In contrast, no such ladder-like LAMP pattern was detected using gel electrophoresis in the absence of target DNA (lane N of Figure 2A). This indicates that the designed TF LAMP primers are suitable for amplifying the target nucleic acid of TF rapidly under isothermal condition. Alternatively, instead of using gel electrophoresis, the LAMP products can be directly visualized after adding SYBR Green I to the reaction mixture when the sample undergoes UV excitation (Figure 2B). The fluorescence assay provides the same results as DNA electrophoresis.

### 2.3. Specificity and Sensitivity of LAMP for Authentication of T. formosanum

**Figure 3:**
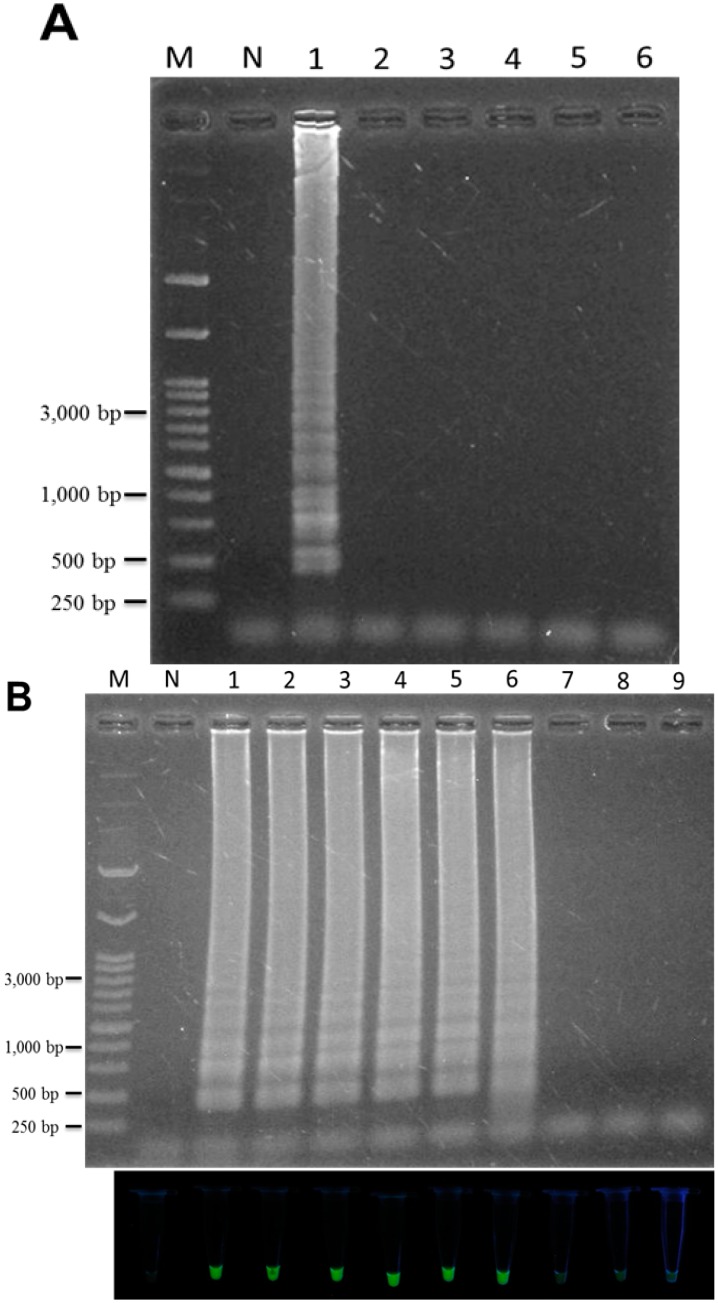
Specificity of the LAMP assay for the authentication of *T. formosanum* (**A**). The LAMP reactions were performed using specifically designed LAMP primers of TF. Lane M, 1 kb of DNA marker; lane N, without plant genomic DNA; lanes 1, *Taraxacum formosanum*; lane 2, *Taraxacum officinale*; lane 3, *Ixeridium laevigatum*; lane 4, *Youngia japonica*; lane 5, *Ixeris chinensis*; lane 6, *Emilia sonchifolia* var. *javanica*; Sensitivity analysis of the LAMP assay for the authentication of *T. formosanum* (**B**). The LAMP reaction was performed using various quantities of genomic TF DNA as template. The upper panels represent LAMP products detected by DNA electrophoresis; the lower panels represented LAMP products detected by SYBR Green I staining and UV excitation. Lane 1, 100 ng; lane 2, 20 ng; lane 3, 1 ng; lane 4, 100 pg; lane 5, 10 pg; lane 6, 1 pg; lane 7, 100 fg; lane 8, 10 fg; lane 9, 1 fg.

**Figure 4:**
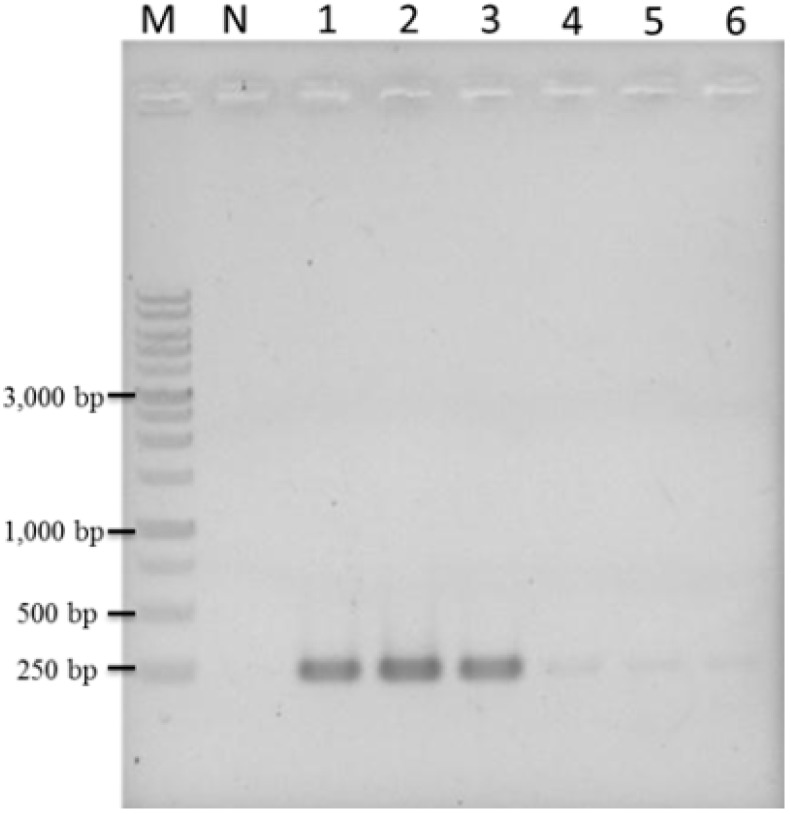
Determination of the PCR sensitivity using species-specific primers. The species-specific primers, F3 and B3, were used with genomic DNA from *Taraxacum formosanum* for a PCR reaction. The PCR reactions were performed after adding various amounts of *Taraxacum formosanum* genomic DNA. Lane M, 1 kb of DNA ladder marker; lane 1, 100 ng; lane 2, 20 ng; lane 3, 1 ng; lane 4, 100 pg; lane 5, 10 pg; lane 6, 1 pg, of extracted genomic DNA was added; Lane N, without TF genomic DNA.

To assess the specificity of LAMP, TF genomic DNA and genomic DNA from the five adulterant plants were used as template. The specificity of the LAMP assay for authentication of TF is shown in Figure 3A. When the LAMP primers were used to test DNA amplification, no amplification in non-targeted adulterant plant DNA was observed. However, when TF genomic DNA was present, the ladder-like LAMP DNA pattern was detected (Figure 3, lane 1). Thus the LAMP method developed in this study is able to discriminate TF from its common adulterants and this can be done rapidly under isothermal conditions within 45 min when SYBR Green dye is added. To evaluate the sensitivity of the LAMP assay when authenticating TF genomic DNA, the varying amounts of extracted TF genomic DNA were used as template DNA. The amount of TF DNA ranged from 100 ng to 1 fg. After performing the LAMP reaction, the ladder-like DNA pattern was observed even when only 1 pg of TF DNA was added to the reaction mixture (Figure 3). No LAMP product was detected by DNA electrophoresis when <1 pg of template DNA was added to the LAMP reaction. The sensitivity results were confirmed using the SYBR Green I fluorescence assay (Figure 3B, lower panel). Therefore, the sensitivity of the LAMP for authenticating TF developed in this study is 1 pg. In contrast, when traditional species-specific PCR reaction was used to detect genomic TF DNA template using two specific designed oligonucleotides F3 and B3 as PCR primer, approximately a 250 bp of PCR product was observed. As to its sensitivity, however, no presence of any PCR product was observed when <1 ng of template TF DNA was added to the PCR reaction (Figure 4); thus the sensitivity is significantly lower than that of LAMP. These results suggest that LAMP is a highly specific and sensitive method for detection of TF genomic DNA.

### 2.4. Identification of T. formosanum within a Mixed Sample

**Figure 5:**
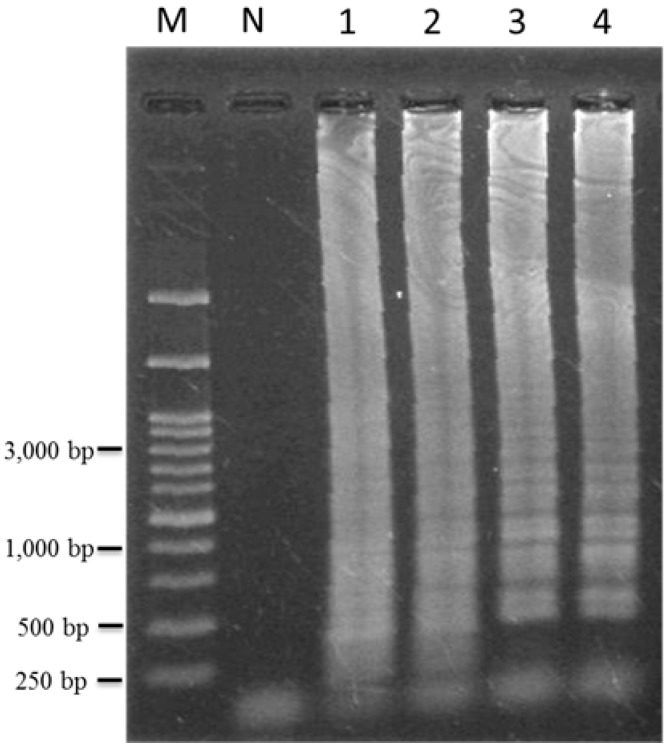
Identification of *Taraxacum formosanum* contaminated with various ratios of adulterant’s genomic DNA using the LAMP method. The genomic DNA of *T. formosanum* and non-target DNA (genomic DNA of *Ixeris chinensis*) were mixed in the ratio 1:10 (lane 4), 1:50 (lane 3) and 1:100 (lane 2), respectively. Lane M, DNA marker; lane N, negative control (genomic DNA of *Ixeris chinensis*); lane 1, 130 ng genomic DNA of each species, *Taraxacum officinale*, *Ixeridium laevigatum*, *Youngia japonica*, *Ixeris chinensis* and *Emilia sonchifolia* var. *javanica*, were all mixed with 130 ng genomic DNA of *T. formosanum* into one sample.

To identify the *T. formosanum* within a mixed sample, multiple adulterant plants mixed into one sample were used as a contamination test. The genomic DNA of *T. formosanum* and non-target DNA (genomic DNA of *Ixeris chinensis*) were mixed in the ratio 1:100, 1:50 and 1:10 (where “1” represents 1 ng), respectively. After LAMP amplification, positive results were detected in all three of the different ratios of mixed samples (Figure 5, lane 2 to lane 4). These DNA diluents demonstrated that the DNA of *T. formosanum* may be amplified using TF LAMP primers when an increase from 10 to 100 ng of non-target DNA was added in reaction mixtures. Additionally, when 130 ng genomic DNA of *Taraxacum officinale*, *Ixeridium laevigatum*, *Youngia japonica*, *Ixeris chinensis* and *Emilia sonchifolia* var. *javanica* were mixed with 130 ng genomic DNA of *T. formosanum* into one sample, the LAMP product of *T. formosanum* was amplified using TF LAMP primers (Figure 5, lane 1). As a result, the developed LAMP method for TF identification was found to be selective and reliable also when DNA of *T. formosanum* is contaminated with genetic material of an adulterant plant.

### 2.5. Application of LAMP to the Authentication of T. formosanum from an Herbal Market

**Figure 6:**
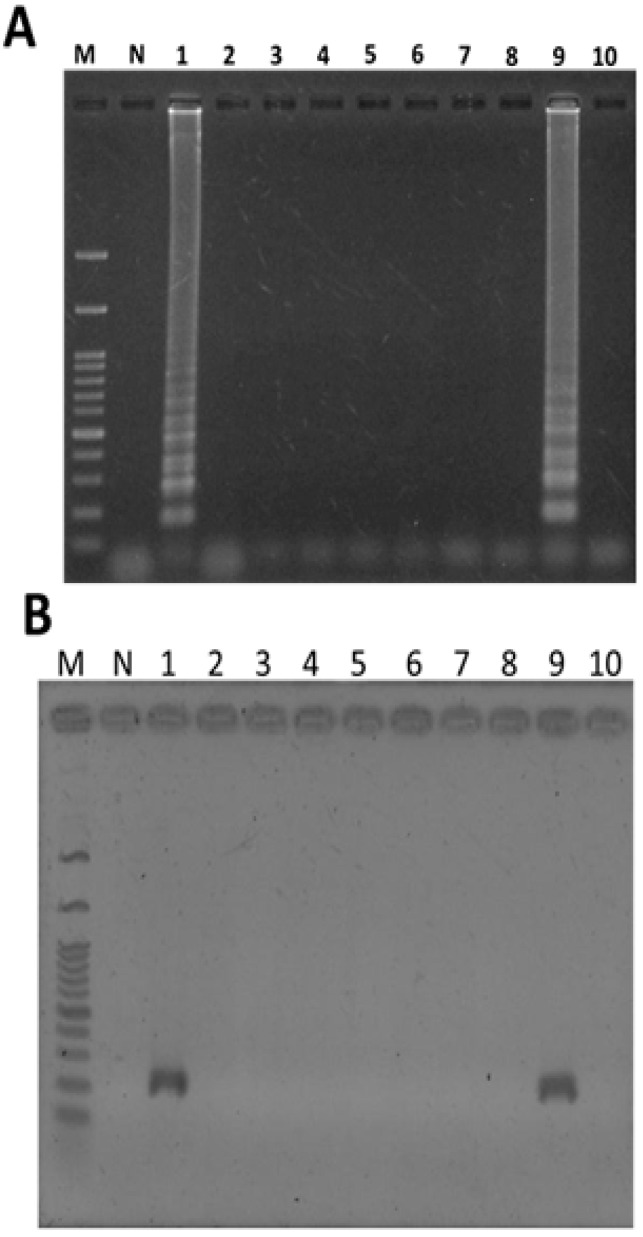
Diagnostic authentication of commercial plants of *Taraxacum formosanum* from various herbal markets using LAMP method (**A**) and PCR (**B**). Ten plants of *Taraxacum formosanum* obtained from various herbal markets were used for rapid authentication using the LAMP method after adding plant genomic DNA. Lane M, 1 kb of DNA ladder marker; lane N, genomic DNA of *Ixeris chinensis*; lane 1–10, ten commercial plant of *Taraxacum formosanum* from various herbal markets.

Based on the above results, this method was evaluated for use as a practical tool by authenticating ten commercially purchased TF plants from different herbal markets in Taiwan. Using genomic DNA extracted from these herbal samples as template, the LAMP product could be amplified from only two of the ten market samples of TF plants within 45 min under isothermal conditions (Figure 6A, lane 1 and lane 9). This result was also confirmed by PCR method (Figure 6B, lane 1 and lane 9). This result indicates that 80% of commercial TF samples are obviously misrepresented.

## 3. Discussion

In this study, we have successfully developed a DNA-based LAMP assay for the rapid, sensitive and specific authentication/identification of TF. The LAMP primers were designed based on a specific DNA region from the ITS2 of TF. In this context, ITS1 showed marginally less sequence divergence across the *Taraxacum* species when ITS1 (4.70%) and ITS2 (4.88%) were compared. Therefore, the conservative DNA regions of ITS2 were aligned and the interspecies/intraspecies variation of the ITS2 sequence between TF and its adulterant plants was chosen when designing the specific LAMP primers (Figure 1). The LAMP primers included two outer primers (F3 and B3) and two inner primers (FIP and BIP). In principle, the LAMP amplicon will be amplified when all of LAMP primers annealed well to their target sequences. Therefore, in the presence of single nucleotide differences, the specificity is still extremely high with LAMP primers [32]. In terms of our primer design (Table 1), one and two specific nucleotides, respectively, located at the 3' ends of F3 and B3, were designed into the two outer primers for the initiation step of the LAMP reaction. In addition, in terms of self-primed DNA synthesis, the two inner primers, FIP and BIP, were also designed to contain one and two specific nucleotides, respectively, located at the 5' ends of FIP and BIP, which are important to the cycling amplification step of LAMP. When the above two key steps are performed, the LAMP will have high specificity and produce a DNA ladder-like pattern when TF DNA is present (Figure 3A).

In terms of sensitivity, the LAMP assay, compared to PCR, PCR-RFLP, RAPD or amplification-refractory mutation system (ARMS), does not require large amounts of template DNA to perform the reaction. In our work, it was sufficient to amplify the DNA when only 1 pg TF DNA was used, representing just 0.001% of the total DNA present extracted from 1 g of plant sample. Nonetheless, the presence of sufficient DNA shortens the total reaction time and speeds up the LAMP reaction. In addition, optimal LAMP primers design can also improve the sensitivity of amplification reaction, if needed [7].

At present, few methods are available for the direct authentication of TF. We have shown here that a PCR assay can also be used to identify TF material and also shows high selectivity. As shown in Figure 4, an abundant DNA fragment of about 250 bp was amplified and detected by agarose gel electrophoresis when TF genomic DNA was added to the PCR reaction. However, the PCR assay needs at least three steps and takes 2 to 3 h. In contrast, the LAMP assay catalyzed by *Bst* DNA polymerase and using the enzyme’s strand-displacement activity is more time-effective than the PCR assay under isothermal condition (65 °C). The LAMP reaction process has no denaturation step, which is different from conventional PCR [1], and is therefore faster. As to previous report, the LAMP reaction was performed within 15 to 60 min under isothermal condition [33]. Therefore, this established LAMP is capable to amplify TF-specific DNA target within 45 min. This will be very useful when making accurate authentications of herbal samples from wild sources or herbal markets. In addition, conventional PCR assays need an accurately controlled thermocycling system and LAMP assays need no such expensive system, only a temperature controlled waterbath or heating block. In addition, high purity/quality of template DNA is required to perform the PCR because PCR-inhibiting agent can be present in the sample to block the PCR reaction. The LAMP reaction, however, is able to amplify tiny amount of template DNA and also performs well without DNA purification [15,33,34,35]. In the pharmaceutical industry, because of the high frequency of adulteration of TF in herbal markets, there is a need to establish easy to operate and reproducible methods for the rapid authentication of such medicinal materials in order to protect the consumer. However, it is important to point out that the LAMP method does not pinpoint cases where a herbal sample in marketplace has been adulterated with other medicinal plants. In Taiwan, *T. officinale*, *I. laevigatum*, *Y. japonica*, *I. chinensis* and *E. sonchifolia* var. *javanica* are commonly substituted for *T. formosanum*. In one study, *I. chinensis* was found to be substituted for TF in 94.7% of samples from herbal markets [36]. The results of our limited market survey are very similar to the previous results and support the practical use of LAMP for the authentication of TF purchased from herbal markets. In addition, some of above samples without LAMP pattern may not have amplified due to the degradation of DNA, over processing of the material or aged samples of the medicinal plant.

## 4. Experimental Section

### 4.1. Plant Samples

The plant samples of *Taraxacum formosanum*, *Taraxacum officinale*, *Ixeridium laevigatum*, *Youngia japonica*, *Ixeris chinensis*, and *Emilia sonchifolia* var. *javanica* were collected from the China Medical University (CMU) medicinal plant garden (Taichung, Taiwan), and were identified by Professor Chao-Lin Kuo of the CMU. The plant voucher specimens were deposited at the department of Chinese pharmaceutical science and Chinese medicine resources of CMU.

### 4.2. DNA Extraction

Total genomic DNA from dried leaves of *Taraxacum formosanum*, *Taraxacum officinale*, *Ixeridium laevigatum*, *Youngia japonica*, *Ixeris chinensis*, and *Emilia sonchifolia* var. *javanica* were extracted by the Geneaid genomic extraction mini kit (Geneaid, Taipei, Taiwan) according to the manufacturer’s instructions [33]. The concentration of total plant genomic DNA obtained was determined by spectrophotometer (NanoVue™, GE Healthcare, Piscataway, NJ, USA). Samples were stored at −20 °C until required.

### 4.3. ITS Sequence Alignment

For ITS sequence alignment, the ITS regions of *Taraxacum formosanum*, *Taraxacum officinale*, *Ixeridium laevigatum*, *Youngia japonica*, *Ixeris chinensis*, and *Emilia sonchifolia* var. *javanica* were obtained from GenBank. The GenBank accession numbers of *Taraxacum formosanum*, *Taraxacum officinale*, *Ixeridium laevigatum*, *Youngia japonica*, *Ixeris chinensis*, and *Emilia sonchifolia* var. *javanica* used in this study were AY862577, AY862576, AY862582, AY862580, AY862578 and EU057987, respectively. The sequence alignment was carried out by software Clustal W2 (http://www.ebi.ac.uk/Tools/msa/clustalw2). The intraspecific variation and interspecific divergence sequence of the ITS2 within *Taraxacum formosanum* and its adulterants was aligned as illustrated in Figure 1.

### 4.4. Design of LAMP Primers

The designs of the LAMP primers (F3, B3, FIP and BIP) for authenticating TF were based on the ITS2 sequence of TF and its adulterants using Primer Explorer V3 software (http://primerexplorer.jp; Eiken Chemical Co., Ltd., Tokyo, Japan). Table 1 and Figure 1, respectively, show the sequence and target positions of the primers.

### 4.5. LAMP Reaction

The LAMP reaction was performed in a reaction mixture, as described previously [33]. Briefly, the reaction mixture contained 1× Bst DNA polymerization buffer, 8 U of Bst DNA polymerase (New England Biolabs, Frankfurt, Germany), 0.2 μM F3 and B3 primers each, 1.5 μM FIP and BIP primers each, and 0.2 mM dNTPs each. Finally, the different amount of plant genomic DNA was added to each LAMP reaction. The mixtures were incubated at 65 °C for 45 min in a heating block (DNA engine, Biorad, CA, USA).

### 4.6. Detection of LAMP Product

The LAMP product was detected using DNA electrophoresis and observation of fluorescence illumination, as described in a previous study [33]. The LAMP products were directly detected by observing the color change at the end of the LAMP reaction. The diluted stock SYBR Green I reagent (Invitrogen, CA, USA), 1:10,000, was added to the LAMP reaction buffer following the LAMP reaction.

### 4.7. PCR

The extracted genomic DNA of *Taraxacum formosanum* was used as template in the polymerase chain reaction (PCR) mixture. PCR was carried out in a reaction mixture containing 1× Pro-taq buffer (10 mM Tris-HCl, 50 mM KCl, 0.01% gelatin, 1.5 mM MgCl_2_, 0.1% Triton X-100, pH 9.0), 0.4 mM each dNTPs, 5 pmole each of forward primer F3 and reverse primer B3, 1U Pro-taq™ DNA polymerase (Protech, Taipei, Taiwan). The conditions used for the PCR was 95 °C for 5 min, followed by 35 cycles of 95 °C for 1 min, 55 °C for 1 min, and 72 °C for 1 min, and a final extension cycle at 72 °C for 10 min. The PCR product was separated by 2% agarose gel electrophoresis and detected by observation of the presence of visible DNA bands after ethidium bromide staining.

## 5. Conclusions

In summary, the LAMP assay developed in the present study is rapid, sensitive and specific for identification of TF plants compared to common TF adulterants. Our LAMP assay approach could become a powerful tool for screening botanic origin and a model for rapid authentication of other herbal resources. 
